# Collision Carcinoma Involving Small Cell Neuroendocrine Carcinoma and Squamous Cell Carcinoma of the Ureter: A Case Report and Review of the Literature

**DOI:** 10.3389/fonc.2021.663119

**Published:** 2021-07-05

**Authors:** Sheng Xu, Lei Xu, Peng Cao, Shiyun Yao, Tingming Wu, Xinming Hu, Hualei Chen, Jun Gu, Xianping Che

**Affiliations:** ^1^ Department of Urology, The Second Affiliated Hospital of Hainan Medical University, Haikou, China; ^2^ Department of Pathology, The Second Affiliated Hospital of Hainan Medical University, Haikou, China

**Keywords:** neuroendocrine carcinoma, squamous cell carcinoma, ureteral, collision carcinoma, small cell

## Abstract

**Background:**

Small cell neuroendocrine carcinoma (SCNEC) of the ureter is a rare tumour, accounting for less than 0.5% of all ureteral tumours. SCNEC tumours are highly aggressive and patients have a poor prognosis. Ureteral SCNEC colliding with other pathological types of tumours is extremely rare. In this paper, we present the case of a patient with ureteral small cell carcinoma colliding with squamous cell carcinoma and review the literature regarding the clinicopathological features, treatment and prognosis of thus tumour. To the best of our knowledge, this is the second identified case of ureteral SCNEC colliding with SCC.

**Case Presentation:**

A 64-year-old male patient presented with a history of 1 month of gross haematuria and 3 months of left flank pain. CT urography revealed a soft tissue mass in the upper ureter, which was slightly enhanced on contrast-enhanced CT. Nephroureterectomy was performed after the patient was diagnosed with a tumour in the left ureter. Microscopy and immunohistochemical examination confirmed the mass to be a SCNEC collision with SCC. Two months after the surgery, the patient received adjuvant chemotherapy (cisplatin/etoposide). After 14 months of follow-up, no local recurrence or distant metastasis was found.

**Conclusion:**

Ureteral collision carcinoma with SCNEC predominantly occurs in Asian individuals, is difficult to diagnose preoperatively and is highly invasive. The current management of ureteral collision carcinoma is a comprehensive treatment based on surgery.

## Introduction

Neuroendocrine carcinoma of the genitourinary tract is extremely rare and is the third most common extrapulmonary site after the gastrointestinal tract and pancreas. Neuroendocrine carcinoma originating from the ureter accounts for 0.5% of all ureteral carcinomas (1). Primary SCNEC of the ureter is even rarer. Ureteral SCNECs are highly aggressive and associated with poor patient prognosis. The incidence of this disease is higher in Asian countries than in other countries. Collision carcinoma is commonly defined as two or more malignant tumours that exist simultaneously in the same organ and have different histological morphologies. We report the case of a patient with a ureteral collision tumour consisting of SCNEC and SCC. Here, we systematically review the literature on ureteral collision carcinoma involving SCNEC and summarize the clinicopathological features, current practice of management and prognosis of this rare tumour (2–15).

## Case Presentation

**Figure 1:**
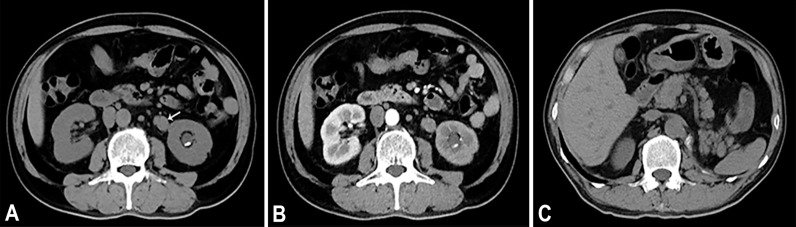
**(A)** Axial unenhanced CT image demonstrates a soft tissue mass (arrow) in the upper of the left ureter. On unenhanced CT scan, ureteral mass measures 37 HU. **(B)** Axial contrast enhanced CT in the arterial phase reveals heterogeneous enhancement of the lesions. On contrast enhanced CT scan, ureteral mass measures 55 HU. **(C)** Axial unenhanced CT image of 7 months after operation.

**Figure 2:**
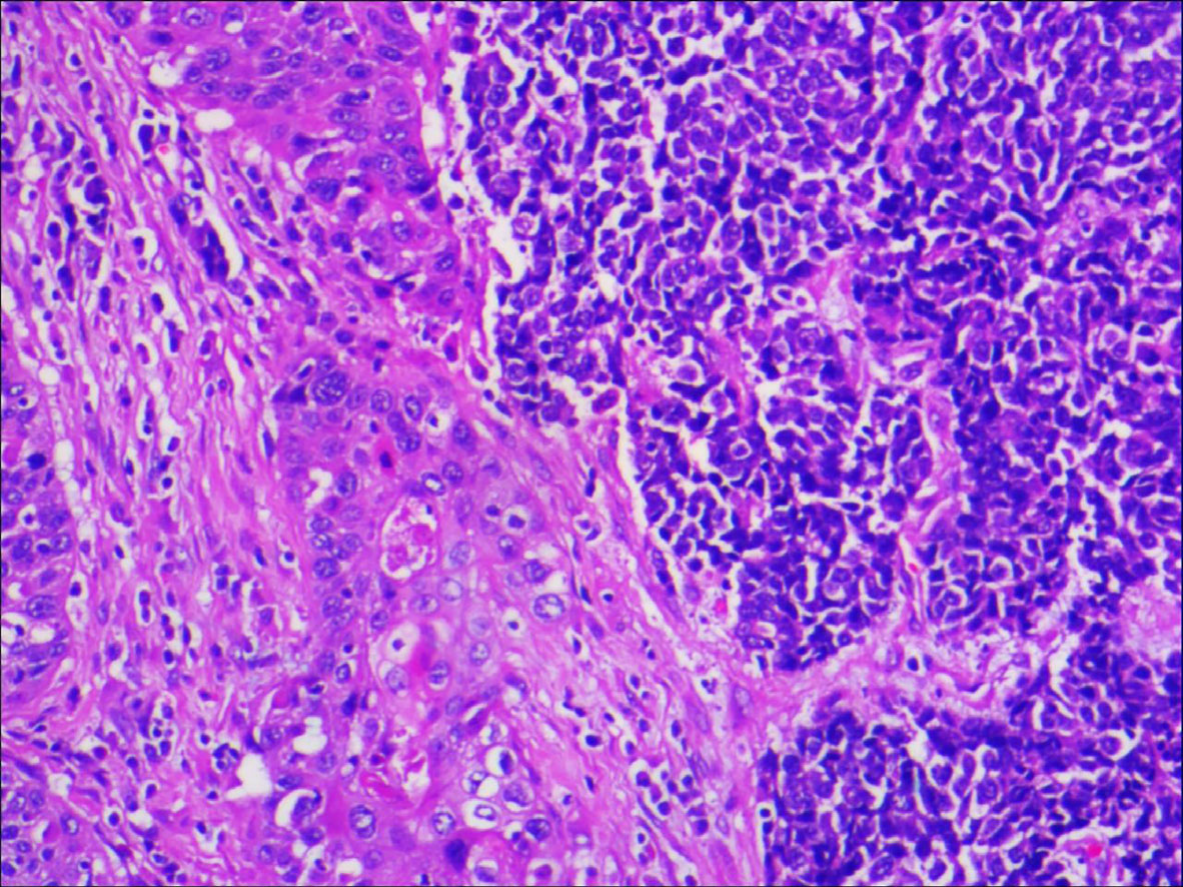
Microscopic examination confirmed that the ureteral tumour cells were composed of two parts, and each component accounted for half of it (magnification, x100).

**Figure 3:**
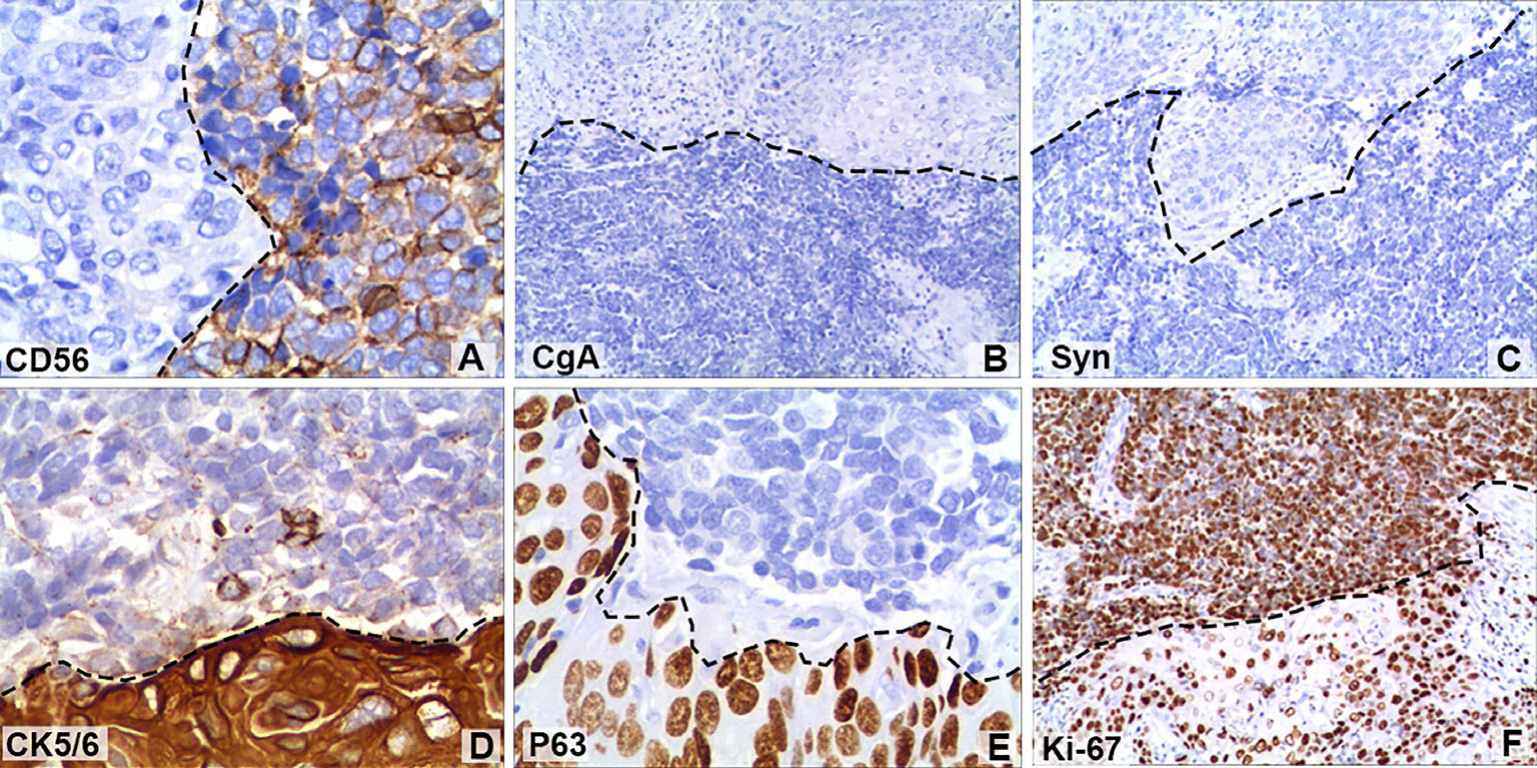
Immunohistochemical staining of some tumour cells showed that **(A)** cluster of differentiation 56 (CD56; magnification, x400) was positive, but **(B)** chromogranin A (CgA; magnification, x100) and **(C)** synaptophysin (Syn; magnification, x100) were negative. Positive immunohistochemical staining for **(D)** cytokeratin 5/6 (CK5/6; magnification, x400) and **(E)** regulator protein 63 (P63; magnification, x400). **(F)** The Ki-67 (magnification, x100) index of neuroendocrine tumour cells was 90%, while that of squamous cell tumours was 40%. The black dotted lines show the boundary between neuroendocrine tumours and squamous carcinomas.

A 64-year-old male patient presented at our hospital with a 1-month history of gross haematuria and a 3-month history of left flank pain. The patient had a 40-year history of smoking and a 1-year history of hypertension. Laboratory examination revealed that the patient’s plasma tumour marker test was negative, and gross haematuria was found in the urine analysis. No tumour cells were detected in the urine by cytological analysis. The CT urogram revealed a soft tissue mass in the upper ureter approximately 1.5 cm from the left ureteropelvic junction (**Figure 1A**), which was slightly enhanced by contrast-enhanced CT (**Figure 1B**). There was mild hydronephrosis in the ureter and the left kidney above the mass, and a stone approximately 7 mm in size was found in the lower calyx of the left kidney. No enlarged lymph nodes were found in the retroperitoneal cavity. A chest CT scan revealed no primary or metastatic lesions in either lung. The whole-body bone scan showed no evidence of metastatic bone disease. No neoplastic lesions were found in the bladder or bilateral ureteral orifice by cystoscopy. The left ureteral lesions were diagnosed as primary ureteral malignant tumours, with a clinical stage of T2N0M0. We performed a laparoscopic left nephroureterectomy for this patient. The review of the pathology was performed independently by two experienced urological pathologists. Gross examination of the resection specimen demonstrated a 1.7×1.5×1.0 cm firm, nodular, grey-white mass in the left ureteral wall, protruding into the lumen. Microscopic examination revealed that the tumour was composed of two types of tumour cells (**Figure 2**). Some tumour cells were densely arranged, with similar cell sizes and less cytoplasm. Frequent mitosis and necrosis were common findings, and tumour thrombi could be seen in the blood vessels. The immunohistochemical results showed the tumour was positive for CD56 and negative for CgA and Syn (**Figure 3**). In the other area of the tumour, cells were arranged in sheets, with varying nuclei size. Cellular atypia was noted, with abundant eosinophilic cytoplasm and obvious keratinization. Immunohistochemically, this portion of tumour cells was positive for CK5/6 and p63. The Ki-67 index of the neuroendocrine tumour cells was 90%, and the Ki-67 index of the squamous cell tumour cells was 40%. The ratio of neuroendocrine tumour cells to squamous cell tumours was approximately 1:1, and the histomorphology and immunophenotype of the tumour cells did not overlap.

**Figure 4:**
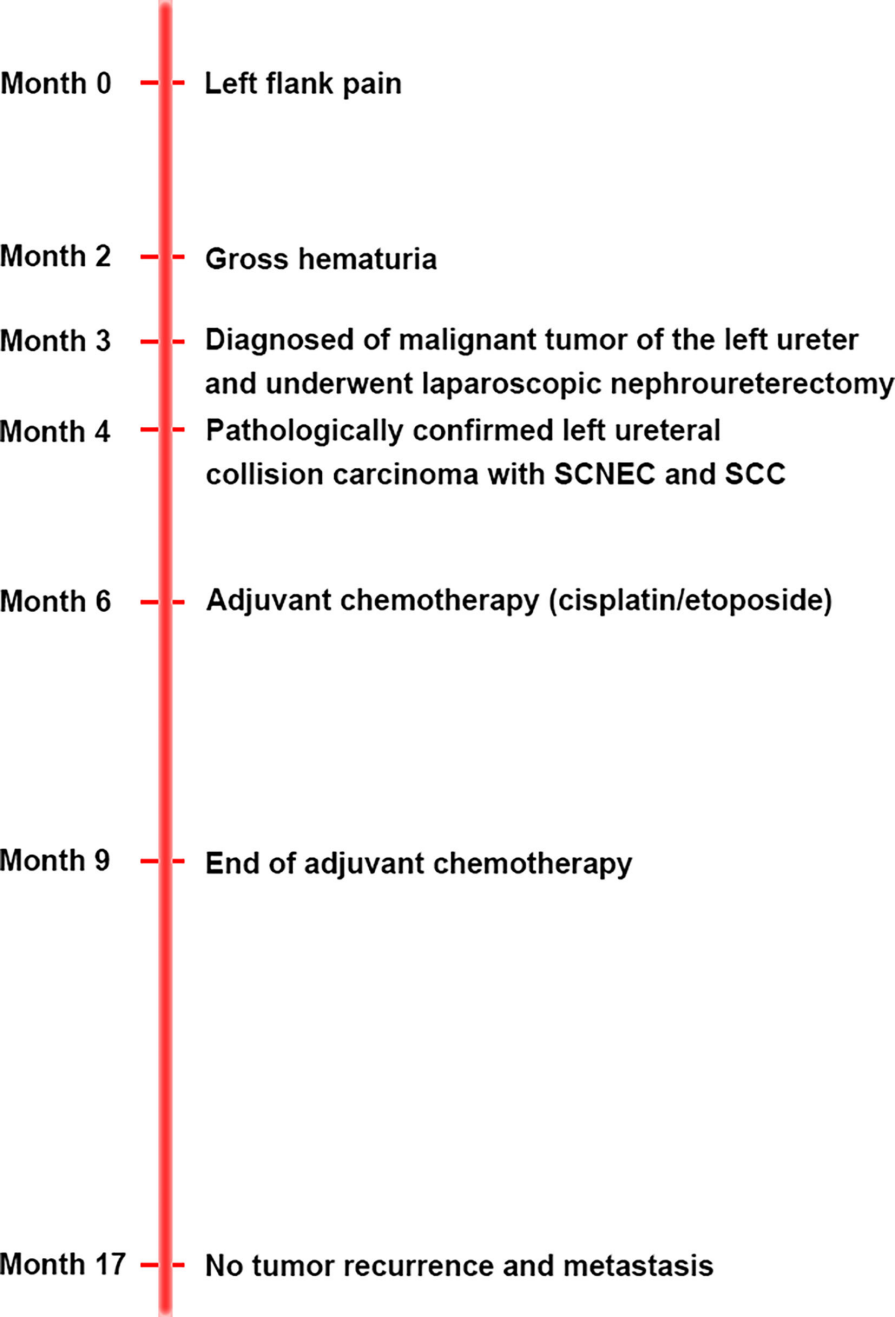
The clinical timeline of the patient.

The patient was diagnosed with ureteral SCNEC colliding with SCC, and the pathological stage was upgraded to T3N0M0. The patient’s postoperative recovery period was uneventful. Two months after the operation, the patient received adjuvant chemotherapy (cisplatin and etoposide every 3 weeks for 4 cycles). No local recurrence or distant metastasis was found during the 14-month follow-up after the operation (**Figure 1C**). **Figure 4** depicts the clinical timeline of the patient.

## Discussion

The most common sites of SCNEC in the genitourinary region are the bladder and prostate (16). However, the incidence of primary ureteral SCNEC is extremely low, accounting for less than 0.5% of all ureteral carcinomas (12). Histologically, the tumour cells of SCNEC are small, densely arranged and have scant cytoplasm. They usually exhibit both frequent mitosis and necrosis. Immunohistochemically, the tumours are often positive for neuroendocrine markers, such as CD56, CgA, and Syn. The dominant features of SCNEC include aggressiveness and poor patient prognosis.

Collision tumours are a rare tumour phenomenon in which two or more types of tumour occur simultaneously in the same organ, and there is no mixing between the different types of tumour components (17). The pathogenesis of ureteral collision carcinoma is unclear. At present, the following three possible pathogeneses have been proposed: 1). the simultaneous proliferation of tumour cells from two different cell lines, 2). multipotent progenitor stem cells that differentiate into two different types of tumour cells and 3). inflammatory cells in the microenvironment of primitive tumour cells accelerate the deterioration of adjacent second tumour cells (18, 19). However, none of these potential mechanism have been substantiated. Ureteral collision carcinoma accounts for approximately half of all SCNEC patients (15). In our case, histological examination showed that the tumour was composed of SCNEC and SCC, and the boundary between the two components of the tumour was clear. Thus, we diagnosed the patient with collision carcinoma.

Ureteral collision carcinoma is challenging for pathologists to diagnose, as it is very rare. Collision carcinomas are heterogeneous, with different patient prognoses and different responses to therapies. From a clinical perspective, identifying the small cell carcinoma of this ureteral collision carcinoma is important for therapeutic and prognostic purposes, as the small cell carcinoma component has a more aggressive nature and is more likely to determine the patient’s outcome. Under light microscopy, small cell carcinoma is composed of small, round or oval cells, with prominent nuclei, scant cytoplasm, and granular chromatin. A high mitotic index can be observed (1, 6, 16). In controversial cases, it is important to confirm the expression of neuroendocrine markers, such as CD56, NSE, Syn, and Chg, by immunohistochemical staining (6, 15).

A systematic review of the English literature revealed only 16 previous cases of ureteral SCNEC colliding with other types of tumours. Including our patient, 17 cases of this rare tumour have been reported to date. The clinicopathological features and prognosis of these 17 patients are shown in **Table 1**. Most of the reported patients are from Asian countries. Approximately 75% of the patients were male, with an average age of 66 years. The main symptoms are haematuria and flank pain. The location of the tumour is common in the lower ureter. There were 15 cases of concurrent SCNEC and urothelial carcinoma. Only one case of collision carcinoma with SCNEC and SCC has been reported. In addition, 2 cases of collisional carcinoma with more than three pathologic types have been reported. According to the TNM classification, postoperative pathologic stage T1-2 tumours accounted for a minority (17.6%) of the tumours, and the majority (82.4%) were T3-4 stage tumours. Due to the early occurrence of haematuria or flank pain, only 3 patients with ureteral collision carcinoma had preoperative lymph node metastases. To date, there has been no report of preoperative distant metastasis.

**Table 1:** Clinicopathological characteristics, staging, treatment, and prognosis of ureteral collision carcinoma with small cell carcinoma.

Author	Age	Sex	Race	Size	Symptom	Location	Pathology	Stage	Surgery	chemotherapy	Radiotherapy	Follow-up months	Outcome
Tsutsumi (2)	60	M	A	NA	GH	Right/upper	UC, Sq, S	pT3N0M0	NU	cPt+ Et (4 cycl),	RT (50 Gy)	16	AWR
Kim (3)	60	M	A	3.8	FP	Right/lower	UC, Sq	pT2N0M0	NU	No	No	36	NED
Chuang (4)	57	M	A	NA	GH;FP	NA	UC	pT3N1M0	NU	No	No	17	DOD
	50	M	A	NA	GH	NA	UC	pT3N0M0	NU	No	No	55	NED
Ryu (5)	78	M	A	5	GH;FP	Right/lower	UC	PT3N1M0	U	No	No	3	NED
Kozyrakis (6)	78	F	NoA	1.7	GH	Right/lower	UC, Sq	pT3N0M0	NU	No	No	6	DOD
Ouzzane (7)	70	M	NoA	0.5	As	Right/lower	UC	pT2N0M0	U	No	No	82	NED
Miller (8)	73	F	NoA	NA	As	NA	UC	pT3NxM0	NU	No	No	10	DOD
Jang (9)	59	M	A	3.5	GH	center/lower	UC	pT3N0M0	NU	cPt+ Et (4 cycl),	RT (54 Gy)	10	NED
Osaka (10)	70	M	NoA	2.6	FP	center/middle	UC	pT2N0M0	NU	Neo;cisPt+irinotecan (3 cycl)	No	38	NED
Nakasato (11)	73	M	A	NA	GH	Right/lower	UC	pT3N0M0	NU	cPt+ Et (2 cycl)	No	12	DOD
Farci (12)	79	F	NoA	2	GH;FP	Right/lower	UC	pT3N1M0	U	Et (1 cycl)	No	5	DOD
Rupert (13)	72	M	NoA	NA	GH	Right/lower	UC	pT3NxM0	NU	PTX(15 cycl)	No	22	AWR
Zhu (14)	47	F	A	15	GH	Right/middle-lower	Sq	pT3N0M0	NU	No	No	5	NED
Zhong (15)	62	M	A	10.5	As	center/upper	UC,S, Ly	PT3N0M0	NU	Yes	No	4	DOD
	56	M	A	2.5	GH	Right/lower	UC,S	PT3N0M0	NU	No	No	6	DOD
Our case	64	M	A	1.7	GH;FP	center/upper	Sq	pT3N0M0	NU	cPt+ Et (4 cycl),	No	14	NED

M, male; F, female; A, Asian; NOA, Non-Asian; FP, flank pain; GH, gross hematuria; As, asymptomatic; UC, urothelial carcinoma; Sq, squamous cell carcinoma; S, sarcoma; Ly, lymphoma; NU, nephroureterectomy; U, ureterectomy; RT, radiotherapy; cPt, carboplatin; Et, etoposide; cisPt, cisplatin; PTX, paclitaxel; Neo, neoadjuvant chemotherapy; DOD, died of disease; NED, non evidence of disease; NA, data not available; AWR, alive with recurrence.

The treatment of SCNEC-based ureteral collision cancer is comprehensive and involves surgical resection (11). Because the clinical manifestation of ureteral collision carcinoma is similar to that of urothelial carcinoma (12) and the size of the specimen taken by ureteroscopy before the operation is small, it is difficult to diagnose SCNEC before the operation. However, Osaka et al. (10) reported the case of a patient with a preoperative diagnosis of ureteral SCNEC who received neoadjuvant chemotherapy. Postoperative pathology showed that ureteral SCNEC collided with urothelial carcinoma. The patient underwent nephroureterectomy after three cycles of cisplatin/irinotecan neoadjuvant chemotherapy. There was no recurrence or metastasis after 38 months of follow-up, which was much longer than the median survival time of patients with ureteral collision carcinoma. The surgical methods reported in the literature are nephroureterectomy and partial ureterectomy. Nephroureterectomy was performed in 14 cases, and partial ureterectomy was carried out only in 3 cases. Adjuvant therapy includes chemotherapy and radiotherapy (7). To our knowledge, only 2 patients received radiotherapy. One patient with sarcoma developed distant metastasis after 16 months of follow-up. Because collision carcinoma contains more than two types of tumour, adjuvant chemotherapy is usually selected according to the tumour with the higher malignant pathological type. The adjuvant chemotherapy regimens reported in the literature were predominantly composed of platinum, including 5 patients who received platinum-based chemotherapy and 2 patients who received nonplatinum-based chemotherapy. Survival time was defined as the time from the date of diagnosis to the date of death or the date of the final follow-up. The 1- and 3-year survival rates were analysed using the Kaplan-Meier method. The median follow-up of the 17 patients was 10 months (range 3-82 months), and the 1-year and 3-year survival rates were 66.6% and 48.6%, respectively. The 3-year survival rates for patients who underwent surgery alone and those who underwent surgery plus chemotherapy were 42.9% and 56.3%, respectively. Mansfield et al. (20) reported that the addition of atezolizumab to carboplatin and etoposide significantly improved the progression-free survival and overall survival rates of patients with extensive-stage small cell lung cancer. However, no report on immunotherapy for ureteral small cell collisional carcinoma has been published.

## Conclusion

Ureteral collision cancer is an extremely rare and aggressive disease. The preoperative diagnosis of ureteral collision carcinoma is difficult, and conclusive diagnosis often relies on pathological examination, particularly immunohistochemistry. The current recommended management approach for ureteral collision carcinoma is a comprehensive treatment strategy involving surgery.

## Data Availability Statement

The original contributions presented in the study are included in the article/supplementary material. Further inquiries can be directed to the corresponding author.

## Ethics Statement

The studies involving human participants were reviewed and approved by the ethics committee of the Medical Faculty of the Second Affiliated Hospital of Hainan Medical University. The patients/participants provided their written informed consent to participate in this study. Written informed consent was obtained from the individual(s) for the publication of any potentially identifiable images or data included in this article.

## Author Contributions

SX, LX, and XC performed the majority of the study. SX, SY, and PC collected relevant pictures. SX, PC, SY, and XC wrote the manuscript. LX, SX, XH, TW, HC, and JG revised the manuscript. All authors contributed to the article and approved the submitted version.

## Conflict of Interest

The authors declare that the research was conducted in the absence of any commercial or financial relationships that could be construed as a potential conflict of interest.
